# Developmental arrest in *Drosophila melanogaster* caused by mitochondrial DNA replication defects cannot be rescued by the alternative oxidase

**DOI:** 10.1038/s41598-018-29150-x

**Published:** 2018-07-18

**Authors:** Ana Paula C. Rodrigues, André F. Camargo, Ana Andjelković, Howard T. Jacobs, Marcos T. Oliveira

**Affiliations:** 1 Departamento de Tecnologia, Faculdade de Ciências Agrárias e Veterinárias, Universidade Estadual Paulista “Júlio de Mesquita Filho”, 14884-900 Jaboticabal, SP Brazil; 2 Faculty of Medicine and Life Sciences and Tampere University Hospital, University of Tampere, Tampere, FI-33014 Finland; 3 Institute of Biotechnology, University of Helsinki, Helsinki, FI-00014 Finland

## Abstract

The xenotopic expression of the alternative oxidase AOX from the tunicate *Ciona intestinalis* in diverse models of human disease partially alleviates the phenotypic effects of mitochondrial respiratory chain defects. AOX is a non-proton pumping, mitochondrial inner membrane-bound, single-subunit enzyme that can bypass electron transport through the cytochrome segment, providing an additional site for ubiquinone reoxidation and oxygen reduction upon respiratory chain overload. We set out to investigate whether AOX expression in *Drosophila* could counteract the effects of mitochondrial DNA (mtDNA) replication defects caused by disturbances in the mtDNA helicase or DNA polymerase γ. We observed that the developmental arrest imposed by either the expression of mutant forms of these enzymes or their knockdown was not rescued by AOX. Considering also the inability of AOX to ameliorate the phenotype of *tko*^*25t*^, a fly mutant with mitochondrial translation deficiency, we infer that this alternative enzyme may not be applicable to cases of mitochondrial gene expression defects. Finding the limitations of AOX applicability will help establish the parameters for the future putative use of this enzyme in gene therapies for human mitochondrial diseases.

## Introduction

Mutations in the human genes *TWNK* and *POLG*, respectively encoding the mitochondrial replicative DNA helicase Twinkle and the catalytic subunit of the mitochondrial replicase pol γ, are associated with diverse human diseases, as well as with aging. In particular, they underlie cases of Progressive External Ophthalmoplegia (PEO) and Alper’s syndrome, for which mitochondrial DNA (mtDNA) depletion, deletions and/or point mutations are frequently observed^1–4^. Twinkle and pol γ are essential components of the mtDNA replication machinery. Twinkle hydrolyzes nucleotide tri-phosphate to unwind the parental double-stranded DNA, providing the single-stranded DNA template for pol γ. In concert with the mitochondrial single-stranded DNA-binding protein (mtSSB), Twinkle and pol γ, and possibly replisome proteins that remain to be identified, can promote mtDNA replication and guarantee proper levels of this genome for correct organellar gene expression^5,6^. mtDNA carries genes that are essential for ATP production via the process of oxidative phosphorylation (OXPHOS).

Mitochondrial diseases exhibit a broad clinical spectrum, often requiring treatments that are specific to each type of disorder, with only a few such treatments shown thus far to be convincingly effective^7^. However, the fact that OXPHOS is often affected in mitochondrial diseases does provide a possible common target for therapeutic interventions: the respiratory chain (RC). Four of the RC complexes transfer electrons to molecular oxygen, a process coupled to energy transduction forming a proton gradient across the mitochondrial inner membrane, whereas a fifth complex uses this gradient to phosphorylate ADP to ATP. Most organisms possess branches of the electron transfer system known as alternative pathways, which are absent in humans, other vertebrates, and insects^8,9^. These pathways may represent “safety valves” to alleviate stress arising from RC overload, as is the case in mitochondrial diseases, preventing excess formation of reactive oxygen species (ROS) and allowing reoxidation of primary electron carriers such as NADH. The alternative oxidase (AOX), for example, is a single-subunit enzyme able to transfer electrons from ubiquinol to oxygen, bypassing RC complexes III and IV, without proton pumping. It has been suggested that AOX expression in human mitochondria could be a treatment for patients with mitochondrial diseases, as a bypass therapy^7,10^.

The transgenic expression of the AOX gene from *Ciona intestinalis* (Tunicata: Ascidiacea) in cultured human cells, in the mouse, and in the fruitfly *Drosophila melanogaster* showed several benefits, which are consistent with a role for AOX in buffering OXPHOS stresses, caused by chemical, genetic or physiological damage. It promoted resistance to inhibitors of complexes III and IV^11–14^, alleviated the metabolic abnormalities of complex IV-deficient cells derived from patients^15^ and the deleterious phenotypes of complex IV insufficiency in *Drosophila*^16^. AOX function has also been reported to provide some benefit in other models of mitochondrial disorders, such as fly lines with knockdown of pol γ^17^ or with mutations in *dj-1β*, the homologue of the human Parkinson’s disease gene *DJ1*^12^, and in *sesB*, the homologue of another PEO gene, *ANT1*^18^. In addition, the rescue by AOX of *Drosophila* models of Alzheimer’s disease and mid-line closure defects suggests that RC dysfunction and/or excess ROS production are not only involved, but also appear in early stages of these pathogeneses^19,20^.

We set out to investigate if the beneficial effects of AOX expression in higher metazoans is applicable to fly lines with mtDNA replication defects. Considering the earlier finding that AOX can ameliorate the phenotype of pol γ-depleted flies, which are defective in mtDNA replication, we hypothesized that AOX could have the same effect in flies with defects in the *Drosophila* homologue of Twinkle. Our data shows that AOX does not rescue developmental lethality, nor alter the biochemical and molecular parameters associated with Twinkle and pol γ mutations or knockdown. This restricts the potential therapeutic use of AOX to specific types of mitochondrial dysfunction.

## Results

### AOX does not rescue lethality caused by Twinkle mutations

Using standard genetic crosses, we initially created 12 fly lines, in which we combined the AOX variant constructs *UAS-empty*^2nd^, *UAS-mutAOX*^2nd^, *UAS-wtAOX*^*8*.*1*^ and *UAS-wtAOX*^*F6*^, with the Twinkle variant constructs *UAS-Twinkle WT*, *UAS-Twinkle K388A*, and *UAS-Twinkle A442P* (see Supplementary Table S1). *UAS-empty*^2nd^ carries the *UAS* promoter without any associated transgene, serving as a promoter dilution control, whereas *UAS-mutAOX*^2nd^ bears the coding sequence for a mutated, inactive form of AOX (mut AOX)^21^. *UAS-wtAOX*^*8*.*1*^ and *UAS-wtAOX*^*F6*^, when induced, lead to the expression of AOX at low and high levels, respectively^12,21^. The Twinkle variant constructs guide the respective overexpression of the wild-type form of the mtDNA helicase (WT), an active-site mutant (K388A), and a PEO mutation-bearing variant (A442P)^22^. The latter represents a fly mutation homologous to the one found in human PEO families, A475P^23^.

**Figure 1:**
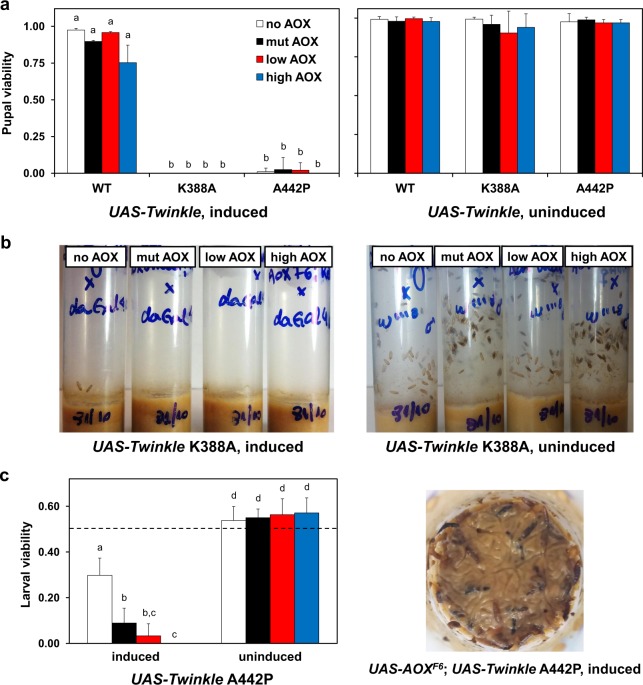
AOX does not rescue the lethality caused by the expression of mutant Twinkle proteins. (**a**) Pupal viability was measured as the mean ratio between the number of adults eclosed and the number of pupae per vial (+/− standard deviation), upon expression (using the *daGAL4* driver, *left panel*) or not (using the *w*^1118^ control, *right panel*) of the indicated AOX and Twinkle variants. (**b**) A representative developmental assay showing the inability of larvae overexpressing Twinkle K388A to enter the pupal stage: whereas the control adult flies at day 7–9 after egg laying (*right panel*) were starting to eclose, Twinkle K388A flies were still alive as active larvae on the food, with very few individuals reaching pre-pupa and dying. (**c**) Larval viability of the *UAS-Twinkle A442P* lines was measured as the mean ratio between the number of wild-type pupae and the total number of pupae per vial (+/− standard deviation), originated from crosses using *UAS-Twinkle A442P* / TM6B-*Tubby* and *daGAL4* (induced) or *w*^1118^ (uninduced). The expected Mendelian ratio of wild-type pupae (carrying *UAS-Twinkle A442P*) for these crosses is 0.5 (dashed line). See Supplementary Fig. S1 for details of the genetic crosses. no AOX, mut AOX, low AOX and high AOX indicate respectively the constructs *UAS-empty*^2nd^, *UAS-mutAOX*^2nd^, *UAS-wtAOX*^*8*.*1* ^^21^ and *UAS-wtAOX*^*F6* ^^12^. Letters a-d indicate significantly different statistical classes (*P* < 0.05) according to one-way ANOVA, followed by the Tukey *post-hoc* test, applied separately for the data in (**a**) *left panel*, and (**c**). No significant differences were found (*P* > 0.05) for the data shown in (**a**) *right panel*.

We then crossed the *UAS-AOX; UAS-Twinkle* lines with the ubiquitous GAL4 driver *daGAL4* (see Supplementary Fig. S1 for crossing schemes, and Supplementary Fig. S2 for immunoblot detection of the overexpressed enzymes). Consistent with previously published data^22^, overexpression of Twinkle WT in the offspring did not affect development, whereas almost all flies expressing the K388A and A442P variants died at the larva L3 or pupal stages (Fig. 1). The lethality of K388A was more severe, as very few larvae were able to enter the pre-pupa stage, dying immediately (Fig. 1b); most L3 larvae were apparently fully developed and mobile at day 5 after egg laying, but remained in this state for an additional 9 days before perishing. Although a very low percentage of adult individuals expressing Twinkle A442P did eclose, indicating that most flies died at the pupa stage, we also observed lethality at the larval stage, judged by the ratio between wild-type and *Tubby* pupae resulting from the cross *UAS-empty*^2nd^*; UAS-Twinkle A442P*/TM6B*-Tubby* X *daGAL4* (Fig. 1c and Supplementary Fig. S1), which was lower than the expected Mendelian proportion.

Coexpression of AOX with Twinkle variants in different combinations had only minor effects on adult eclosion rates, and AOX did not rescue the larval lethality caused by Twinkle K388A (Fig. 1a,b). Surprisingly, high levels of functional AOX appeared to intensify the larval lethality caused by Twinkle A442P, as no *UAS-wtAOX*^*F6*^/*2; UAS-Twinkle A442P*/*daGAL4* larva (see Supplementary Fig. S1) ever developed beyond the pre-pupa stage, resembling the more severe K388A phenotype (Fig. 1c). Our data shows that AOX cannot alleviate the developmental disturbances caused by overexpression of Twinkle mutations, and suggests it could in fact aggravate them.

### AOX does not alter mtDNA copy number in mutant Twinkle flies

**Figure 2:**
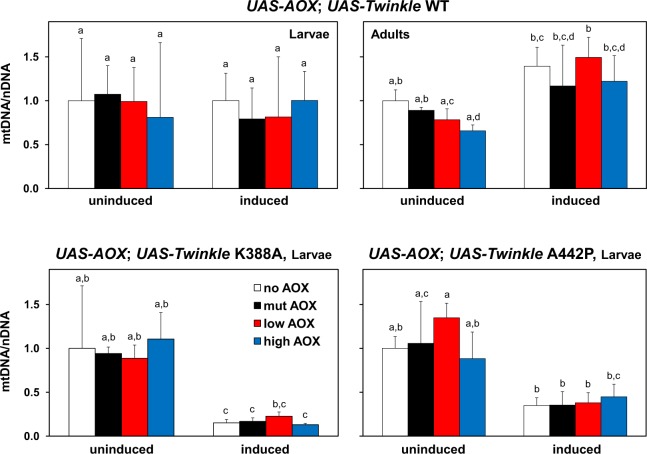
AOX does not interfere with mtDNA copy number in mutant Twinkle lines. Total DNA from larva and adult samples and quantitative PCR were performed as described in the Methods. Uninduced and induced indicate samples originated from the crosses *UAS-AOX*; *UAS-Twinkle* X *w*^*1118*^ and *UAS-AOX*; *UAS-Twinkle* X *daGAL4*, respectively. Relative mtDNA copy number (mtDNA/nDNA) was normalized by the mean 2^−ΔΔCT^ of control samples from the crosses *UAS-empty*^*2nd*^; *UAS-Twinkle* X *w*^*1118*^ in every panel. no AOX, mut AOX, low AOX and high AOX indicate respectively the constructs *UAS-empty*^*2nd*^, *UAS-mutAOX*^*2nd*^, *UAS-wtAOX*^*8*.*1*^ ^21^ and *UAS-wtAOX*^*F6*^ ^12^. Letters a-d indicate significantly different statistical classes (*P* < 0.05) according to one-way ANOVA, followed by the Tukey *post-hoc* test, applied separately for the data in each panel.

In addition to the expected mtDNA depletion caused by the overexpression of the Twinkle mutants^22^, we hypothesized that mtDNA copy number might be affected by AOX, since mtDNA maintenance has been linked to mitochondrial membrane potential^24^ and AOX could potentially lower this parameter by diverting respiratory electron flow. We therefore measured relative mtDNA copy number in our fly lines via quantitative PCR, but found that AOX does not significantly change mtDNA levels in any Twinkle combination (Fig. 2). Overexpressing Twinkle WT did not alter mtDNA copy number in L3 larvae, although a 30–40% increase was observed in adult flies (Fig. 2). Copy number increase upon Twinkle WT overexpression has been reported previously for *Drosophila* adults^22^ and cells in culture^25^, for human cells in culture^26^ and for mice^27,28^. As expected, overexpression of Twinkle K388A and A442P depleted mtDNA in L3 larvae to 20–40% of control levels, independently of AOX expression.

### Developmental effects of Twinkle mutants are tissue-specific

**Figure 3:**
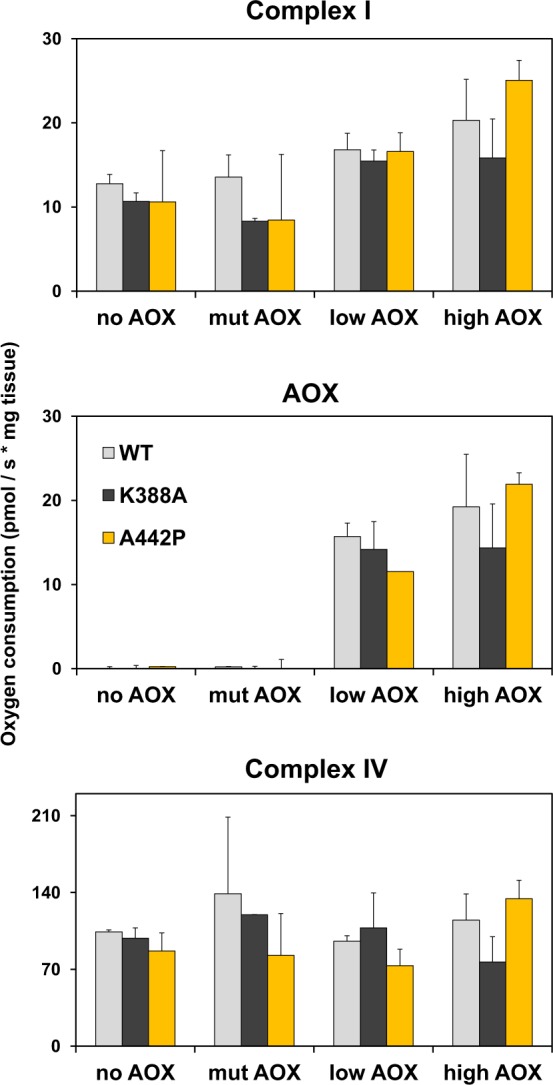
Mitochondrial respiration is not affected by overexpression of Twinkle nor AOX. Oxygen consumption was measured as described in the Methods in samples expressing the indicated AOX and Twinkle variants (*UAS-AOX*; *UAS-Twinkle* X *daGAL4*). Respiration linked to complex I substrate oxidation (*upper panel*) was calculated as the mean oxygen consumption after addition of pyruvate, proline, malate and ADP, followed by inhibition with antimycin A and rotenone. AOX activity (antimycin A-resistant respiration) was calculated as the mean oxygen consumption after addition of pyruvate, proline, malate, ADP and antimycin A, followed by inhibition with propyl-gallate and rotenone. Complex IV activity was calculated as the mean oxygen consumption after addition of TMPD and ascorbate, followed by inhibition with potassium cyanide. no AOX, mut AOX, low AOX and high AOX indicate respectively the constructs *UAS-empty*^*2nd*^, *UAS-mutAOX*^*2nd*^, *UAS-wtAOX*^*8*.*1*^ ^21^ and *UAS-wtAOX*^*F6*^ ^12^. No significant differences were found (*P* > 0.05) according to one-way ANOVA, followed by the Tukey *post-hoc* test.

Next, we checked if AOX could improve mitochondrial function upon expression of the Twinkle mutants, although it cannot rescue the flies from lethality. Surprisingly, none of the Twinkle variants altered mitochondrial oxygen consumption driven by complex I or IV substrate oxidation in the body wall tissues of L3 larvae (Fig. 3 and Supplementary Fig. S3). Even though there was an increase in respiration in all Twinkle overexpressors in the presence of functional AOX, this was not statistically significant, except in the presence of the complex III inhibitor antimycin A, as expected (Fig. 3, *middle panel*). Because the larval body wall consists mostly of muscular and nervous tissues, we tested if tissue-specific expression of the Twinkle mutations using the *mhcGAL4* and *elavGAL4* drivers, respectively, could reproduce the lethal phenotype observed in the presence or absence of AOX. Consistent with the lack of detectable OXPHOS dysfunction in the larval body wall, flies overexpressing Twinkle K388A or A442P in muscles or neurons completed normal development (Supplementary Fig. S4). Again, coexpression of AOX had no effect on the developmental parameters analyzed. In addition to the observation that the L3 larvae expressing K388A and A442P via *daGAL4* induction appear actively “healthy” for several days after the expected time of pupation, our mitochondrial respiration data and developmental assays using *mhcGAL4* and *elavGAL4* suggest that the lethality caused by the Twinkle variants are independent of the action of these mutants in tissues traditionally affected by mitochondrial disorders.

### The knockdown of Twinkle is pupal lethal and cannot be rescued by AOX

To eliminate the possibility that the inability of AOX to rescue our Twinkle models was because of the overexpression system, we also created and tested four *UAS-AOX* lines combined with *UAS-Twinkle RNAi* (see Supplementary Table S1). To our knowledge, this is the first report describing the use of this RNAi line from the Vienna *Drosophila* Research Center (VDRC), so we first verified its effect on the levels of *Twinkle* mRNA in controls (*UAS-Twinkle RNAi*; *UAS-AOX* X *w*^1118^) and induced individuals (*UAS-Twinkle RNAi*; *UAS-AOX* X *daGAL4*) to show the efficacy of the construct (see Supplementary Fig. S1 for crossing schemes). As expected, *Twinkle* mRNA was knocked down to 40–50% of the control levels in all lines (Supplementary Fig. S5a). Flies with low levels of Twinkle presented mtDNA depletion (Supplementary Fig. S5c) and reached the pupal stage, unlike most mutant overexpressors, but ~75% of them failed to eclose. AOX did not significantly alter viability (Supplementary Fig. S5b), except when expressed at a high level, which once again appeared to exacerbate the lethality caused by Twinkle disturbances. This data demonstrates that the AOX bypass does not mitigate the effects of Twinkle defects caused either by overexpression of mutants or by depletion of the endogenous enzyme.

### AOX is unable to rescue other mtDNA replication defects

**Figure 4:**
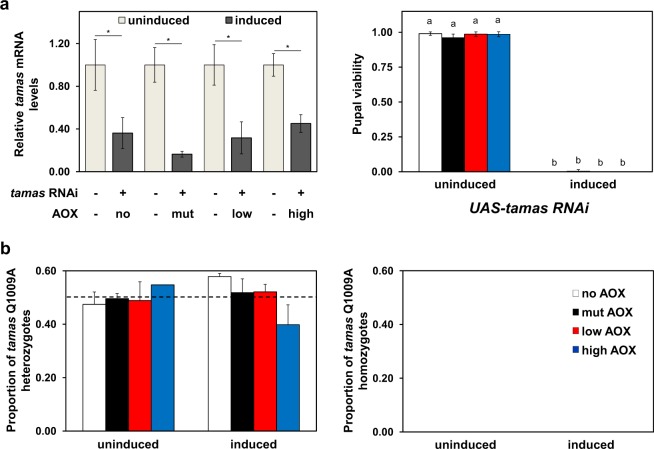
AOX does not rescue the developmental defects caused by genetic disturbances of pol γ. (**a**) Transcript levels of *tamas*, the coding gene for the catalytic subunit of pol γ (*left panel*), were measured as described in the Methods. Pupal viability (*right panel*) was measured as the mean ratio between the number of adults eclosed and the number of pupae per vial (+/− standard deviation). Uninduced and induced indicate samples originated from the crosses *UAS-tamas RNAi*; *UAS-AOX* X *w*^*1118*^ and *UAS-tamas RNAi*; *UAS-AOX* X *daGAL4*, respectively. (**b**) Viability of the *tamas Q1009A* lines was measured as the mean ratio between the number of wild-type adults and the total number of individuals per vial (+/− standard deviation). *Left panel*, progeny originated from crosses using *tamas Q1009A*/CyO; *UAS-AOX* and *w*^*1118*^ (uninduced) or *daGAL4* (induced) to generate a heterozygous *tamas Q1009A* progeny, serving as controls. The expected Mendelian ratio of wild-type adults (carrying one *tamas Q1009A* allele) for these crosses is 0.5 (dashed line). *Right panel*, progeny originated from crosses using *tamas Q1009A*/CyO; *UAS-AOX* and *tamas Q1009A*/CyO (uninduced) or *tamas Q1009A*/CyO; *daGAL4* (induced) to generate a homozygous *tamas Q1009A* progeny. The expected Mendelian ratio of wild-type adults (carrying two *tamas Q1009A* alleles) for these crosses is zero, as *tamas Q1009A* in homozygozity is lethal^29^. See Supplementary Fig. S6 for details of the genetic crosses. no AOX, mut AOX, low AOX and high AOX indicate respectively the constructs *UAS-empty*^*3rd*^, *UAS-mutAOX*^*3rd*^, *UAS-wtAOX*^*7*.*1*^ ^21^ and *UAS-wtAOX*^*F24*^ ^12^. *indicates statistical differences (*P* < 0.05) according to Student’s *t*-tests. Letters a-b indicate significantly different statistical classes (*P* < 0.05) according to one-way ANOVA, followed by the Tukey *post-hoc* test, applied separately for the data in (**a**) *right panel*, and (**b**) *right* and *left panels*. No significant differences were found (*P* > 0.05) for the data shown in (**b**).

Considering that Twinkle and pol γ are part of the same replication machinery, our findings contrast those of Humphrey *et al*.^17^, in which AOX expression in *Drosophila* was shown to counteract deleterious effects of pol γ (*tamas*) knockdown in development (using the ubiquitous *Act5CGAL4* driver) and in neurodegeneration models (using specific neural drivers). We therefore created and analyzed eight additional *UAS-AOX* lines combined with *UAS-tamas RNAi* or with a polymerase-active-site mutant of the catalytic subunit of pol γ, *tamas Q1009A* (see Supplementary Table S1). This RNAi line is the same as was used by Humphrey *et al*.^17^, whereas the *tamas Q1009A* mutation has been engineered in its natural genomic location, presenting the same expression pattern for the gene as in a wild-type line^29^. Both models promoted distinct developmental defects (see Supplementary Figs S1 and S6 for crossing schemes): *UAS-tamas RNAi*, when induced, led to mtDNA depletion (Supplementary Fig. S5d) and ~100% lethality at the pupal stage (Fig. 4a), whereas *tamas Q1009A* individuals died as L3 larvae (Fig. 4b). Coexpression of AOX, at low or high levels, had no effect on the phenotypes of these pol γ-deficient flies (Fig. 4 and Supplementary Fig. S5d), indicating that this alternative enzyme is in general unable to ameliorate mtDNA replication defects caused by disturbances in different components of the mtDNA replisome.

## Discussion

It has been shown that the xenotopic expression of *C*. *intestinalis* AOX in mammalian and insect mitochondria is efficient in rescuing diverse models of mitochondrial dysfunction, especially those with OXPHOS complex IV deficiencies^30,31^. AOX appears to be safely expressed and to function only when the cytochrome segment of the RC is deficient^11,12^, which makes it an attractive enzyme to be used in future therapies for mitochondrial diseases^10^. Nevertheless, there are aspects of AOX expression in heterologous systems that warrant further evaluation, such as the effects of its thermogenic and antioxidant properties. Heat production by mitochondrial uncoupling is to date only detectable in AOX-expressing cells in the presence of antimycin A^32^, but the lowering of ROS apparently occurs even under normal physiological conditions when OXPHOS is properly functioning, at least in adult flies^33^. In addition, the fact that AOX can rescue developmental defects not related, *a priori*, to mitochondrial dysfunction, such as cleft thorax, notched wings, leg malformations, and bristle abnormalities in *Drosophila*^20^, indicates that yet unknown cellular mechanism(s) is(are) operating when AOX is present in the mitochondria of higher animals.

At the other end of the spectrum of AOX applicability, there is a reported case of a true mitochondrial dysfunction, whose associated phenotypes were not ameliorated by the transgenic expression of this alternative enzyme, namely flies bearing the *tko*^*25t*^ mutation^34^. This mutant has a defective mitochondrial ribosomal protein S12, impairing organellar protein synthesis and causing developmental delay and neurological problems^35^. Because another alternative enzyme, the alternative NADH dehydrogenase Ndi1, was also unable to rescue *tko*^*25t*^, the authors concluded that this mutant has a more complex metabolic defect, e.g. affecting multiple RC components, or some other process^34^. Here, we describe another case of AOX inability to rescue mitochondrial dysfunction in *Drosophila*. Overexpression of Twinkle mutants or knockdown of the endogenous enzyme caused developmental lethality at different stages with varying levels of mtDNA depletion, which was not alleviated by AOX. AOX was also unable to rescue the lethality caused by expression of a catalytically inactive pol γ or knockdown of its catalytic subunit, nor any of the molecular and biochemical parameters associated with mutant Twinkle expression. At high levels, AOX was in fact more detrimental for flies with Twinkle defects. One possible explanation is that the mutant Twinkle, or the depletion of the wild-type enzyme, in some way affects the organization of the inner mitochondrial membrane, and the further expression of a membrane-targeted protein (active or inactive AOX) synergistically impairs even more larval/pupal viability. Twinkle has been associated with a specific lipid domain of the inner mitochondrial membrane^36,37^, consistent with this hypothesis.

However, similar aggravations of developmental problems were also observed for *tko*^*25t*^ in the presence of Ndi1, or Ndi1 plus AOX^34^. We speculate that in both cases, the metabolic consequences of severely defective mtDNA replication/mitochondrial translation may result in the activation of AOX, leading to an additional decrease in ATP production due to partial diversion of electron flow. Both the multi-subunit mitochondrial ribosomal machinery, of which the *tko* gene product is an essential part, and the modularly-architectured, multimeric Twinkle enzyme are dependent on the energy of nucleotide tri-phosphate hydrolysis for their proper functions in organellar protein synthesis and genome maintenance, respectively (reviewed in^3,38^). For example, the recombinant human wild-type Twinkle not only requires Mg^2+^ and ATP for dsDNA unwinding and ssDNA translocation, but the enzyme also presents significantly higher stability *in vitro* in the presence of such compounds^39^. Therefore, fluctuations in the ATP level in a sensitive mitochondrial genetic condition, such as that found in the *tko*^*25t*^ and Twinkle models, may worsen the defects caused by such mutant proteins, which may in turn require even higher than normal nucleotide levels to maintain their fragile stability and/or function.

One of the primary effects of disturbing Twinkle or pol γ is the decrease of intact copies of mtDNA (via rearrangements, nucleotide substitutions or degradation)^22,29^, which in turn impairs mitochondrial RNA and protein synthesis^40^. *tko*^*25t*^ produces a defect directly in the mitochondrial translation apparatus, downstream of Twinkle and pol γ, although its phenotypic consequences appear less severe than those of our mtDNA replication models. Nonetheless, the end result is similar: incorrect or insufficient subunits of all four OXPHOS complexes partly encoded in mtDNA, which AOX cannot circumvent. The fact that the mitochondrial genome encodes seven subunits of OXPHOS complex I and two subunits of complex V (~70% of all mtDNA genes) may explain the inability of AOX to rescue mitochondrial gene expression defects, as this enzyme is predicted to counteract complexes III and/or IV deficiencies.

Although in accord with the above hypothesis, our data appears inconsistent with the previously published findings showing significant improvement in the phenotypes associated with the knockdown of pol γ in diverse cell types of *Drosophila*^17^. We speculate that differences in the genetic background of the fly lines, in dietary conditions, and in experimental design might explain these discrepancies. Even though conditions appear to be homogenous within each study, they are difficult to compare side-by-side. Our implementation of two controls (for promoter dilution and for AOX enzymatic activity), plus two expression levels for AOX, and two defective pol γ models (knockdown and mutant expression), does represent a robust experimental design. On the other hand, the use by Humphrey *et al*.^17^ of several neural-specific GAL4 drivers does make their report reliable regarding pol γ depletion in the nervous system. Deficiencies of RC complex III and/or IV in specific neuronal classes are thus implied to underlie neurodegeneration in pol γ disease, in contrast to the broader effects of mtDNA replication defects documented in our study. Another important difference is that the drivers used for ubiquitous knockdown are different: *daGAL4*, used here, and *Act5CGAL4* used by Humphrey *et al*.^17^ may differ in their exact strength, tissue and stage-specificity, and response to conditions. Notably, in combination with *daGAL4*, we found *UAS-tamas RNAi* to be pupal lethal, whereas in combination with *Act5CGAL4*, Humphrey *et al*.^17^ found it to be only semilethal, with AOX impacting the number of progeny. Clearly, the matter needs further investigation.

Our combined biochemical and genetic data also revealed an unexpected phenomenon regarding the overexpression of Twinkle variants in *Drosophila*: the absence of OXPHOS defects in larval body-wall mitochondria despite severe mtDNA depletion in whole L3 larval homogenates, as well as the absence of developmental lethality in the case of tissue-specific expression of mutant Twinkle in muscle and neurons. Maternal protein, RNA and even mtDNA may supply enough mitochondrial activity in those tissues to complete development. Because the large storage of triglycerides in the L3 larvae could interfere with the respiration measurements, we used a previously validated protocol^29^ in which the animals were dissected to eliminate all internal organs and the hemolymph, preserving the larval body wall (comprising muscle, nerve and epidermis) for these experiments. The results imply either that the larval phenotype described by us and others^22^ upon mutant Twinkle overexpression is caused by effects in one or more of these internal organs, or else could be due to an additional but unknown biological role of Twinkle, separate from mtDNA maintenance. The findings suggest a crucial role for mitochondria in the brain, fat body or other tissues that generate and respond to signals governing metamorphosis, over which AOX has no influence. Future studies using other tissue-restricted drivers for the larva, including ring gland, fat body, Malpighian tubule, salivary gland, glia, neuroendocrine cells, among others, will help us understand these roles.

As the human diseases associated with Twinkle and pol γ mutations account for a significant portion of all mitochondrial disorders, AOX may have limited applicability in therapy. Indeed, taking into account the combined data on *tko*^*25t*^, Twinkle and pol γ, we would argue that consideration of the possible use of AOX in therapy should exclude deficiencies in mtDNA maintenance or gene expression, unless clear benefits can be robustly demonstrated in a specific, clinically relevant model.

## Methods

### Fly stocks and maintenance

The standard line *w*^*1118*^, balancer lines CyO, TM3,Sb and TM6B-*Tubby*, and driver lines *daughterless-GAL4* (*daGAL4*), *myosin heavy chain-GAL4* (*mhcGAL4*) and *embryonic lethal abnormal vision*^*C155*^*-GAL4* (*elavGAL4*) were obtained from stock centers. The lines *UAS-AOX*^*F6*^, *UAS-AOX*^*F24* ^^12^, *UAS-wtAOX*^*8*.*1*^, *UAS-wtAOX*^*7*.*1*^, *UAS-empty*^*2nd*^, *UAS-empty*^*3rd*^, *UAS-mutAOX*^*2nd*^ and *UAS-mutAOX*^*3rd* ^^21^ were used in genetic crosses with the balancers described above to establish double transgenic lines in combination with *UAS-Twinkle WT*, *UAS-Twinkle K388A*, *UAS-Twinkle A442P*^22^, *UAS-Twinkle RNAi* (VDRC ID 108644/KK), *UAS-tamas RNAi* (VDRC ID 106955/KK) and *tamas Q1009A*^29^. Supplementary Table S1 shows the genotypes of all lines created for this work. All fly lines were first backcrossed into the *w*^*1118*^ background for six-to-ten generations, and were maintained in standard diet^12^ at 18 or 25 °C, with 12 hr light/dark cycles.

### Developmental assays

10–15 virgin adult females of the indicated genotypes (Supplementary Table S1) were crossed with 5–7 adult control (*w*^*1118*^) or GAL4-expressing males (*daGAL4* or *mhcGAL4*) per vial, and allowed to lay eggs for 24 hrs at 25 °C in standard diet^12^, where the offspring were kept until eclosion (see Supplementary Figs S1 and S5 for crossing schemes). For the neuron-specific transgene expression, *elavGAL4* females were crossed with *UAS* construct-bearing males. The offspring of the *UAS-Twinkle RNAi* lines were kept at 29 °C to enhance the knockdown and its associated phenotype. Development was analyzed visually at the larval stage and quantitatively at the pupal stage, as the mean ratio between the number of eclosed adults and the number of pupae per vial, for a total of 8 vials per experiment, with 3 biological replicates.

### Immunoblotting

Protein extracts from 10 whole 1-to-3-day-old adult males of the indicated genotype were prepared by gentle grinding of tissues in 200 μl PBS containing 1.5% Triton X-100 on ice. The suspension was centrifuged at 16,000 *g*_*max*_ for 10 min at 4 °C, and protein concentration of the supernatant was measured by the Bradford method. 100 μg of total protein extracts were mixed with 5X Laemmli buffer (10% SDS, 50% glycerol, 25% 2-mercaptoethanol, 0.02% bromophenol blue and 0.3125 M Tris-HCl, pH 6.8), denatured at 95 °C for 5 min and resolved by SDS-PAGE on 12% polyacrylamide gels at 120 V for approximately 1.5 h. Proteins were transferred to nitrocellulose membranes using the semi-dry gel transfer system MSD10 (Major Science, USA) for 15 min at room temperature. Membranes were blocked in PBST (8 mM Na_2_HPO_4_, 2 mM KH_2_PO_4_, 150 mM NaCl, 30 mM KCl, 0.05% Tween 20, pH 7.4) containing 5% dried nonfat milk for 2 h at room temperature or overnight at 4 °C. Primary antibodies [rabbit polyclonal anti-AOX^12^ (1:10,000), rabbit polyclonal anti-Dmhelicase^25^ (1:4,000), and mouse monoclonal anti-PDH E1α (1:5,000, Abcam, UK)] were incubated for at least 1 h at room temperature, followed by three washes (10 min each) with PBST. Secondary antibodies [HRP-conjugated goat anti-rabbit and anti-mouse IgGs (1:10,000, Bio-Rad, USA)] were incubated for at least 2 h at room temperature, and washed as described above. Membranes were incubated in Luminol substrate detection system Immun-Star HRP (Bio-Rad, USA), and chemiluminescence signals were detected on a ChemiDoc Imaging System (Bio-Rad, USA).

### Nucleic acid isolation and quantitative PCR

Total DNA was extracted as described previously^24^, using 5–10 L3 larvae (5 days after egg laying) or adult males (1–3 days after eclosion) per preparation. To estimate relative mtDNA copy number, 15 ng of total DNA were used as template in a 20 μl quantitative PCR reaction, containing 10 μl PowerUp SYBR Green Master Mix^TM^ (Thermo Fisher Scientific) and 10 μM of each primer. The primers used were 16S-R (5′-TCGTCCAACCATTCATTCCA-3′) and 16S-L (5′-TGGCCGCAGTATTTTGACTG-3′) to amplify the mitochondrial *16S rRNA* gene, and RpL32F (5′-AGGCCCAAGATCGTGAAGAA-3′) and RpL32A (5′-TGTGCACCAGGAACTTCTTGAA-3′) to amplify the single-copy, nuclear *RpL32* gene. Reactions were performed in a StepOnePlus^TM^ Real-Time PCR instrument (Applied Biosystems), under the manufacturer’s recommended conditions: 95 °C for 2 min, followed by 40 cycles of 95 °C for 3 s and 60 °C for 30 s. The ΔΔC_T_ values were calculated by comparing the ratios between the mitochondrial and nuclear target genes. The relative mtDNA copy number values were derived by comparing each group of *UAS-AOX*; *UAS-Twinkle* lines to the same control sample (*UAS-empty*^*2nd*^; *UAS-Twinkle* X *w*^*1118*^) that was arbitrarily given the value 1.0.

Total RNA was prepared from 5 larvae using the TRIzol® plus PureLink RNA Mini Kit, and was reverse-transcribed using the SuperScript™ IV VILO™ Master Mix with ezDNase™ Enzyme kit (Thermo Fisher Scientific), according to the manufacturer’s recommendations. The quantitative PCR reactions to estimate the relative amounts of *mtDNA-helicase* (Twinkle) and *tamas* transcripts were performed essentially as described above, except that 1 μl of a 1:25 dilution of the reverse transcription reaction was used as template, along with 10 μM of the specific primers: TwinkleF (5′CCAAAGCGGTTCTAGTCAGC3′) and TwinkleR (5′GACTGGCATCGTAGTGCAAC3′) for *mtDNA-helicase*; tamasF (5′AAAATGTTCTCCATCACAAAGG3′) and tamasR (5′TTGGGACGATGAAATACCTC3′) for *tamas*; and RpL32F and RpL32A (described above) for *RpL32*. The relative transcript values were derived from the standard curve method, by comparing the ratio between *mtDNA helicase* and *RpL32*, or *tamas* and *RpL32* for each *UAS-RNAi*; *UAS-AOX* X *daGAL4* line to its control sample (*UAS-RNAi*; *UAS-AOX* X *w*^*1118*^) that was arbitrarily given the value 1.0.

### Mitochondrial oxygen consumption

Five L3 larvae (5 days after egg laying) of the indicated genotype were dissected as previously^29^ in ice-cold PBS, eliminating the internal organs and the hemolymph, to obtain the larval body wall, which was then weighed in an analytical balance, gently homogenized in isolation buffer (250 mM sucrose, 5 mM Tris-HCl, 2 mM EGTA, 1% BSA, pH 7.4), and transferred to the oxygraph O2k chambers (Oroboros, Inc.). Oxygen consumption was measured at 25 °C in assay buffer (120 mM KCl, 0.5 mM KH_2_PO_4_, 3 mM HEPES, 1 mM EGTA, 1 mM MgCl_2_, 1% BSA, pH 7.2) after addition of substrates for complex I (5 mM sodium pyruvate, 5 mM L-proline and 1.5 mM malate), and complex IV (1.9 mM N,N,N′,N′-tetramethyl-p-phenylenediamine [TMPD] and 4 mM ascorbate) in the presence of 1 mM ADP. Complex I- and IV-driven respiration was corrected for the signal obtained after addition of 50 nM antimycin A and 5 nM rotenone, and 100 nM potassium cyanide, respectively. The antimycin A-resistant complex I-driven oxygen consumption, corrected for the signal obtained after addition of 200 nM propyl-galate and 5 nM rotenone, was attributed to AOX.

### Statistical analyses

Statistical differences among the indicated groups of flies were tested using one-way ANOVA with Tukey *post hoc* test, as indicated in the figure legends, except for differences in *Twinkle* and *tamas* transcript levels, for which Student *t*-tests were applied. The analyses were performed using the software SAS University Edition v9.4 (SAS Institute Inc.).

### Data Availability Statement

Any material or fly line used in this work is available from the corresponding author upon individual requests at any moment.

## Electronic supplementary material

Supplementary Information
