# Supplementary material for: Developmental arrest in Drosophila melanogaster caused by mitochondrial DNA replication defects cannot be rescued by the alternative oxidase
Source: Sci Rep. 2018 Jul 18;8:10882. doi: 10.1038/s41598-018-29150-x (PMC6052043; doi:10.1038/s41598-018-29150-x)
Supplement: Supplementary file 1 — Supplementary Information [file 41598_2018_29150_MOESM1_ESM.pdf]

Supplementary Information

Developmental arrest in *Drosophila melanogaster* caused by mitochondrial DNA replication defects cannot be rescued by the alternative oxidase

Ana Paula C. Rodrigues, André F. de Camargo, Ana Andjelković, Howard T. Jacobs and Marcos T. Oliveira

Supplementary Table

Table S1. Genotype of the double transgenic lines created for this work.

	no AOX	non-functional AOX	low levels, functional AOX	high levels, functional AOX
Twinkle WT	*UAS-empty*^{2nd}/*UAS-empty*^{2nd}; *UAS-Twinkle WT*/TM6B	*UAS-mutAOX*^{2nd}/*UAS-mutAOX*^{2nd}; *UAS-Twinkle WT*/TM6B	*UAS-wtAOX*$^{8.1}$/*UAS-wtAOX*$^{8.1}$; *UAS-Twinkle WT*/TM6B	*UAS- wtAOX*^{F6}/*UAS-wtAOX*^{F6}; *UAS-Twinkle WT*/TM6B
Twinkle K388A	*UAS-empty*^{2nd}/*UAS-empty*^{2nd}; *UAS-Twinkle K388A/UAS-Twinkle K388A*	*UAS-mutAOX*^{2nd}/*UAS-mutAOX*^{2nd}; *UAS-Twinkle K388A/UAS-Twinkle K388A*	*UAS-wtAOX*$^{8.1}$/*UAS-wtAOX*$^{8.1}$; *UAS-Twinkle K388A/UAS-Twinkle K388A*	*UAS- wtAOX*^{F6}/*UAS-wtAOX*^{F6}; *UAS-Twinkle K388A/UAS-Twinkle K388A*
Twinkle A442P	*UAS-empty*^{2nd}/*UAS-empty*^{2nd}; *UAS-Twinkle A442P*/TM6B	*UAS-mutAOX*^{2nd}/*UAS-mutAOX*^{2nd}; *UAS-Twinkle A442P*/TM6B	*UAS-wtAOX*$^{8.1}$/*UAS-wtAOX*$^{8.1}$; *UAS-Twinkle A442P*/TM6B	*UAS- wtAOX*^{F6}/*UAS-wtAOX*^{F6}; *UAS-Twinkle A442P*/TM6B
Twinkle RNAi	*UAS-Twinkle RNAi/UAS-Twinkle RNAi*; *UAS-empty*^{3rd}/*UAS-empty*^{3rd}	*UAS-Twinkle RNAi/UAS-Twinkle RNAi*; *UAS-mutAOX*^{3rd}/*UAS-mutAOX*^{3rd}	*UAS-Twinkle RNAi/UAS-Twinkle RNAi*; *UAS-wtAOX*$^{7.1}$/*UAS-wtAOX*$^{7.1}$	*UAS-Twinkle RNAi/UAS-Twinkle RNAi*; *UAS-wtAOX*^{F24}/*UAS-wtAOX*^{F24}
pol γ RNAi	*UAS-tamas RNAi/UAS-tamas RNAi*; *UAS-empty*^{3rd}/*UAS-empty*^{3rd}	*UAS-tamas RNAi/UAS-tamas RNAi*; *UAS-mutAOX*^{3rd}/*UAS-mutAOX*^{3rd}	*UAS-tamas RNAi/UAS-tamas RNAi*; *UAS-wtAOX*$^{7.1}$/*UAS-wtAOX*$^{7.1}$	*UAS-tamas RNAi/UAS-tamas RNAi*; *UAS-wtAOX*^{F24}/*UAS-wtAOX*^{F24}
pol γ Q1009A	*UAS-tamas Q1009A*/CyO-GFP; *UAS-empty*^{3rd}/*UAS-empty*^{3rd}	*UAS-tamas Q1009A*/CyO-GFP; *UAS-mutAOX*^{3rd}/*UAS-mutAOX*^{3rd}	*UAS-tamas Q1009A*/CyO-GFP; *UAS-wtAOX*$^{7.1}$/*UAS-wtAOX*$^{7.1}$	*UAS-tamas Q1009A*/CyO-GFP; *UAS-wtAOX*^{F24}/*UAS-wtAOX*^{F24}

Supplementary Figure Legends

Figure S1. Genetic crosses used to test rescue by AOX of Twinkle mutants, and Twinkle or pol γ knockdown. The double transgenic lines created for this work (always used as females) are described in the Supplementary Table S1. Progeny classes are color-coded to indicate their significance in each experiment. *2* and *3* represent respectively the non-transgenic 2nd and 3rd chromosomes of w^{1118} background. TM6B represents a balancer for the 3rd chromosome, inducing the dominant *Tubby* phenotype in the larvae and pupae, and the dominant *Humeral* phenotype in adults. Individuals not carrying a balancer chromosome morphologically appear as wild-type.

Figure S2. AOX and Twinkle are coexpressed in the *UAS-AOX*; *UAS-Twinkle* lines. **a**, Fly protein samples were processed and immunoblotted as described in the Methods. Induced, *UAS-AOX*; *UAS-Twinkle* X *mhcGAL4*; uninduced, *UAS-AOX*; *UAS-Twinkle* X w^{1118}. no AOX, mut AOX and low AOX indicate respectively the constructs *UAS-empty*^{2nd}, *UAS-mutAOX*^{2nd}, and *UAS-wtAOX*$^{8.1}$ [1], whereas high AOX and high AOX 1/10 indicate the same *UAS-wtAOX*^{F6} contruct[2], except that in the latter the protein sample was diluted 10fold before electrophoresis. The original blots of induced samples processed with the anti-Dmhelicase antibody to detect the ~66 kDa Twinkle (**b**), or with a mix of anti-AOX and anti-PDH E1α to detect the ~36 kDa AOX and the ~40 kDa E1α subunit of pyruvate dehydrogenase (**c**), and the blots of uninduced samples processed with the same antibodies (**d**, **e**) are shown. Red and blue arrows indicate respectively the 75 and 37 kDa markers of the Precision Plus Protein™ Dual Color Standards (Bio-Rad), revealed by a chemiluminescent WesternSure™ pen (Li-Cor). Note that endogenous Twinkle in the uninduced controls was barely detectable, consistent with previously

published work[3], indicating the low abundance of this enzyme. The multiple bands detected by the anti-Dmhelicase antibody are due to cross-reaction with non-mitochondrial proteins, as shown by Matshuima and Kaguni[4]. Note also that the mutant version of AOX was not detected in any *UAS-mutAOX*^{2nd} lines, even when the membrane was overexposed for several minutes (data not shown), although the construct is present in the lines' DNA, as verified by PCR using primers specific to the AOX mutation and sequencing (data not shown).

Figure S3. Mitochondrial respiration in the *UAS-AOX*; *UAS-Twinkle* uninduced control lines. Oxygen consumption was measured as described in the Methods in samples carrying the indicated AOX and Twinkle variants (uninduced, *UAS-AOX*; *UAS-Twinkle* X *w*^{1118}). Respiration linked to complex I substrate oxidation (*upper panel*) was calculated as the mean oxygen consumption after addition of pyruvate, proline, malate and ADP, followed by inhibition with antimycin A and rotenone. AOX activity (antimycin A-resistant respiration) was calculated as the mean oxygen consumption after addition of pyruvate, proline, malate, ADP and antimycin A, followed by inhibition with propyl-gallate and rotenone. Complex IV activity was calculated as the mean oxygen consumption after addition of TMPD and ascorbate, followed by inhibition with potassium cyanide. no AOX, mut AOX, low AOX and high AOX indicate respectively the constructs *UAS-empty*^{2nd}, *UAS-mutAOX*^{2nd}, *UAS-wtAOX*$^{8.1}$ [1] and *UAS-wtAOX*^{F6} [2]. No significant differences were found ($P > 0.05$) according to one-way ANOVA, followed by the Tukey *post-hoc* test.

Figure S4. Overexpression of Twinkle mutants in muscle and neuronal tissues does not cause developmental arrest. Pupal viability was measured as the mean ratio

between the number of adults eclosed and the number of pupae per vial (+/- standard deviation), upon expression of the indicated AOX and Twinkle variants. Expression was driven to the musculature by *mhcGAL4* (mhc>K388A and mhc>A442P), and to the neurons by *elavGAL4* (elav>K388A and elav>A442P). no AOX, mut AOX, low AOX and high AOX indicate respectively the constructs *UAS-empty*^{2nd}, *UAS-mutAOX*^{2nd}, *UAS-wtAOX*$^{8.1}$ [1] and *UAS-wtAOX*^{F6} [2]. No significant differences were found ($P > 0.05$) according to one-way ANOVA, followed by the Tukey *post-hoc* test.

Figure S5. AOX does not rescue the developmental defects caused by Twinkle and tamas knockdown. a, Transcript levels of *Twinkle* were measured as described in the Methods and show the efficiency of the RNAi when induced. * indicates statistical differences ($P < 0.05$) according to Student's *t*-tests. **b**, Pupal viability was measured as the mean ratio between the number of adults eclosed and the number of pupae per vial (+/- standard deviation). **c** and **d**, Total DNA from larva samples and quantitative PCR were performed as described in the Methods. Uninduced and induced indicate samples originated from the crosses *UAS-Twinkle RNAi*; *UAS-AOX* X w^{1118} and *UAS-Twinkle RNAi*; *UAS-AOX* X *daGAL4*, respectively, in **b** and **c**, and from the crosses *UAS-tamas RNAi*; *UAS-AOX* X w^{1118} and *UAS-tamas RNAi*; *UAS-AOX* X *daGAL4*, respectively, in **d**. Relative mtDNA copy number (mtDNA/nDNA) was normalized by the mean $2^{-\Delta\Delta CT}$ of control samples from the crosses *UAS-RNAi*; *UAS-empty*^{3rd} X w^{1118} in each panel. no AOX, mut AOX, low AOX and high AOX indicate respectively the constructs *UAS-empty*^{3rd}, *UAS-mutAOX*^{3rd}, *UAS-wtAOX*$^{7.1}$ [1] and *UAS-wtAOX*^{F24} [2]. Letters a-d indicate significantly different statistical classes ($P < 0.05$) according to one-way ANOVA, followed by the Tukey *post-hoc* test, applied separately for the data in **b**, **c** and **d**.

Figure S6. Genetic crosses used to test rescue by AOX of pol γ Q1009A mutant.

The double transgenic lines created for this work (always used as females) are described in the Supplementary Table S1. Progeny classes are color-coded to indicate their significance in each experiment. *2* and *3* represent respectively the non-transgenic 2nd and 3rd chromosomes of w^{1118} background, and *tamas* represents the coding gene for the catalytic subunit of pol γ. CyO represents a balancer for the 2nd chromosome, inducing the dominant *curly wings* phenotype in the adults, and lethal in homozygosity. Individuals not carrying a balancer chromosome morphologically appear as wild-type.

References

1 Andjelkovic, A. *et al.* Diiron centre mutations in *Ciona intestinalis* alternative oxidase abolish enzymatic activity and prevent rescue of cytochrome oxidase deficiency in flies. *Sci Rep* **5**, 18295, doi:10.1038/srep18295 (2015).

2 Fernandez-Ayala, D. J. *et al.* Expression of the *Ciona intestinalis* alternative oxidase (AOX) in *Drosophila* complements defects in mitochondrial oxidative phosphorylation. *Cell Metab* **9**, 449-460, doi:10.1016/j.cmet.2009.03.004 (2009).

3 Sanchez-Martinez, A. *et al.* Modeling pathogenic mutations of human twinkle in *Drosophila* suggests an apoptosis role in response to mitochondrial defects. *PLoS One* **7**, e43954, doi:10.1371/journal.pone.0043954 (2012).

4 Matshushima, Y. & Kaguni, L.S. Differential phenotypes of active site and human autosomal dominant progressive external ophthalmoplegia mutations in *Drosophila* mitochondrial DNA helicase expressed in Schneider cells. *J Biol Chem* **282**, 9436-9444, doi:10.1074/jbc.M610550200 (2007).

Crosses involving *UAS-Twinkle* WT or A442P:

♀ *UAS-AOX* / *UAS-AOX* ; *UAS-Twinkle* / TM6B X ♂ 2 / 2 ; *daGAL4* / *daGAL4*

Expected ratio of progeny:

0.5	*UAS-AOX* / 2 ; *UAS-Twinkle* / *daGAL4*
0.5	*UAS-AOX* / 2 ; TM6B / *daGAL4*

♀ *UAS-AOX* / *UAS-AOX* ; *UAS-Twinkle* / TM6B X ♂ 2 / 2 ; 3 / 3 (w^{1118})

Expected ratio of progeny:

0.5	*UAS-AOX* / 2 ; *UAS-Twinkle* / 3
0.5	*UAS-AOX* / 2 ; TM6B / 3

Crosses involving *UAS-Twinkle* K388A:

♀ *UAS-AOX* / *UAS-AOX* ; *UAS-Twinkle K388A* / *UAS-Twinkle K388A* X ♂ 2 / 2 ; *daGAL4* / *daGAL4*

Expected ratio of progeny: 1.0 *UAS-AOX* / 2 ; *UAS-Twinkle K388A* / *daGAL4*

♀ *UAS-AOX* / *UAS-AOX* ; *UAS-Twinkle K388A* / *UAS-Twinkle K388A* X ♂ 2 / 2 ; 3 / 3 (w^{1118})

Expected ratio of progeny: 1.0 *UAS-AOX* / 2 ; *UAS-Twinkle K388A* / 3

Crosses involving *UAS-Twinkle RNAi* or *UAS-tamas RNAi*:

♀ *UAS-RNAi* / *UAS-RNAi* ; *UAS-AOX* / *UAS-AOX* X ♂ 2 / 2 ; *daGAL4* / *daGAL4*

Expected ratio of progeny: 1.0 *UAS-RNAi* / 2 ; *UAS-AOX* / *daGAL4*

♀ *UAS-RNAi* / *UAS-RNAi* ; *UAS-AOX* / *UAS-AOX* X ♂ 2 / 2 ; 3 / 3 (w^{1118})

Expected ratio of progeny: 1.0 *UAS-RNAi* / 2 ; *UAS-AOX* / 3

Twinkle and AOX expressors (experimental progeny, induced)

Twinkle non-expressors / AOX expressors (controls, *Tubby* phenotype)

Twinkle or tamas depletors (experimental progeny, induced)

Non-expressors or non-depletors (controls, uninduced)

Supplementary Fig. S1 – Rodrigues *et al*.

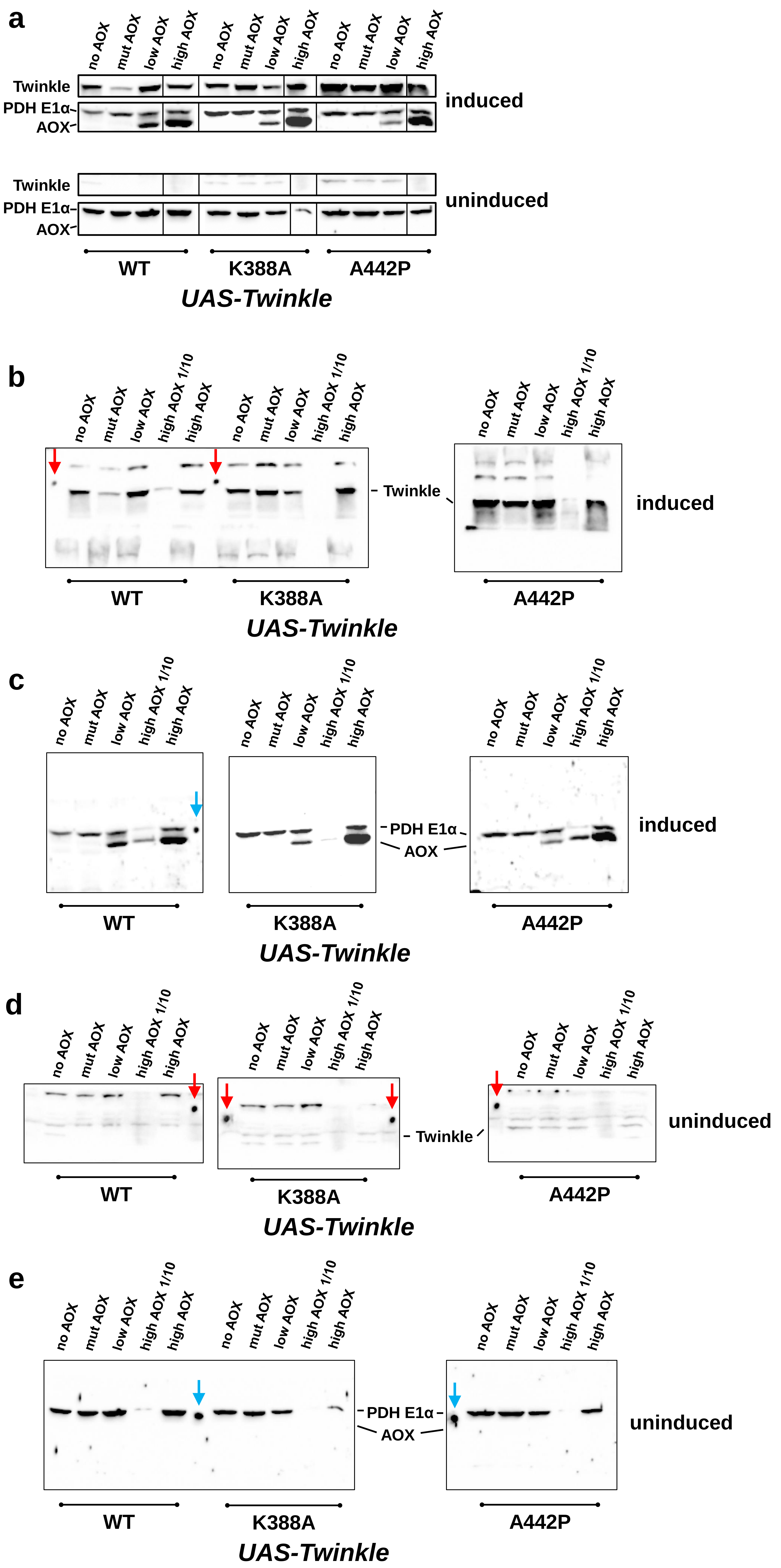

Supplementary Fig. S2 – Rodrigues *et al*.

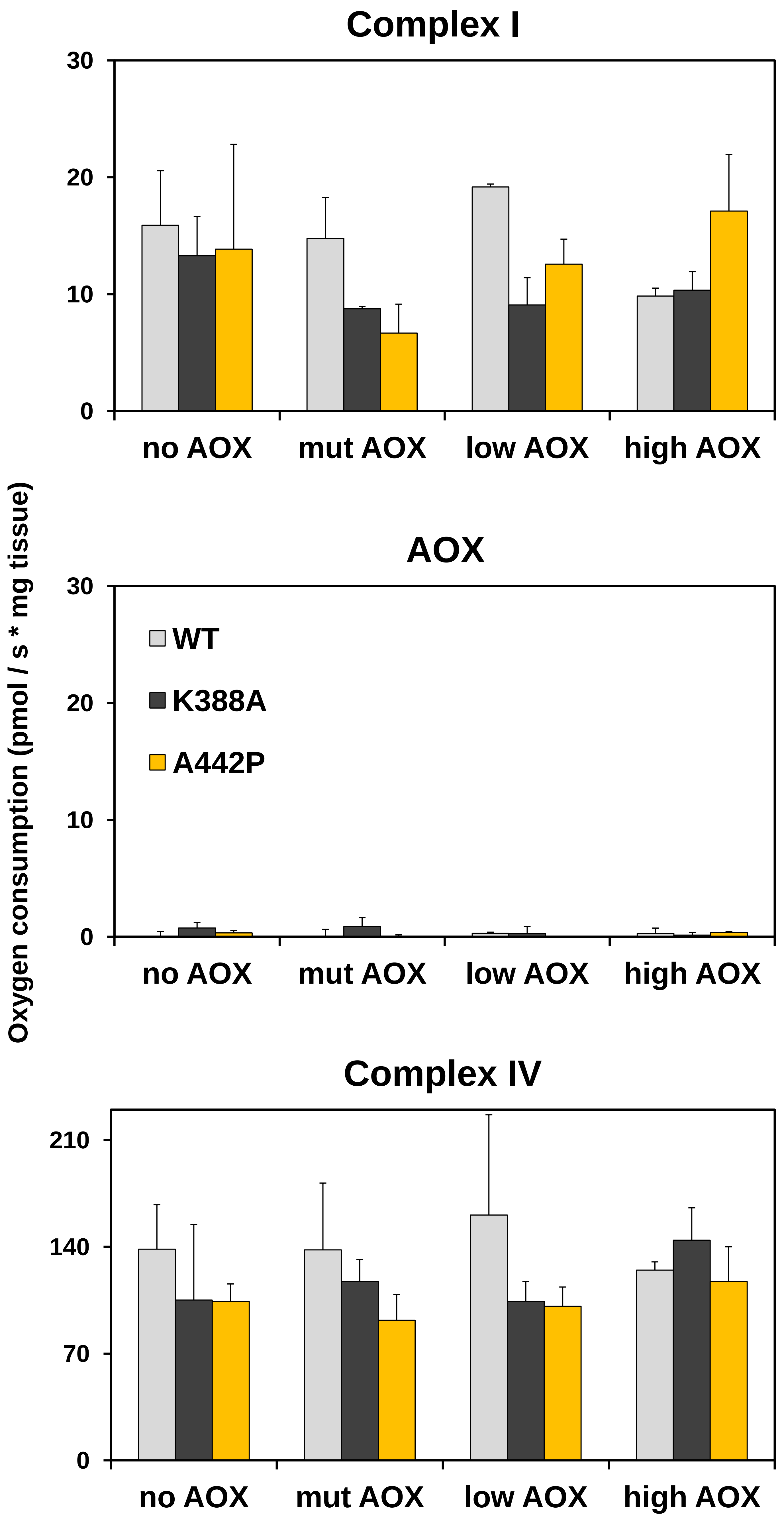

Supplementary Fig. S3 – Rodrigues *et al*.

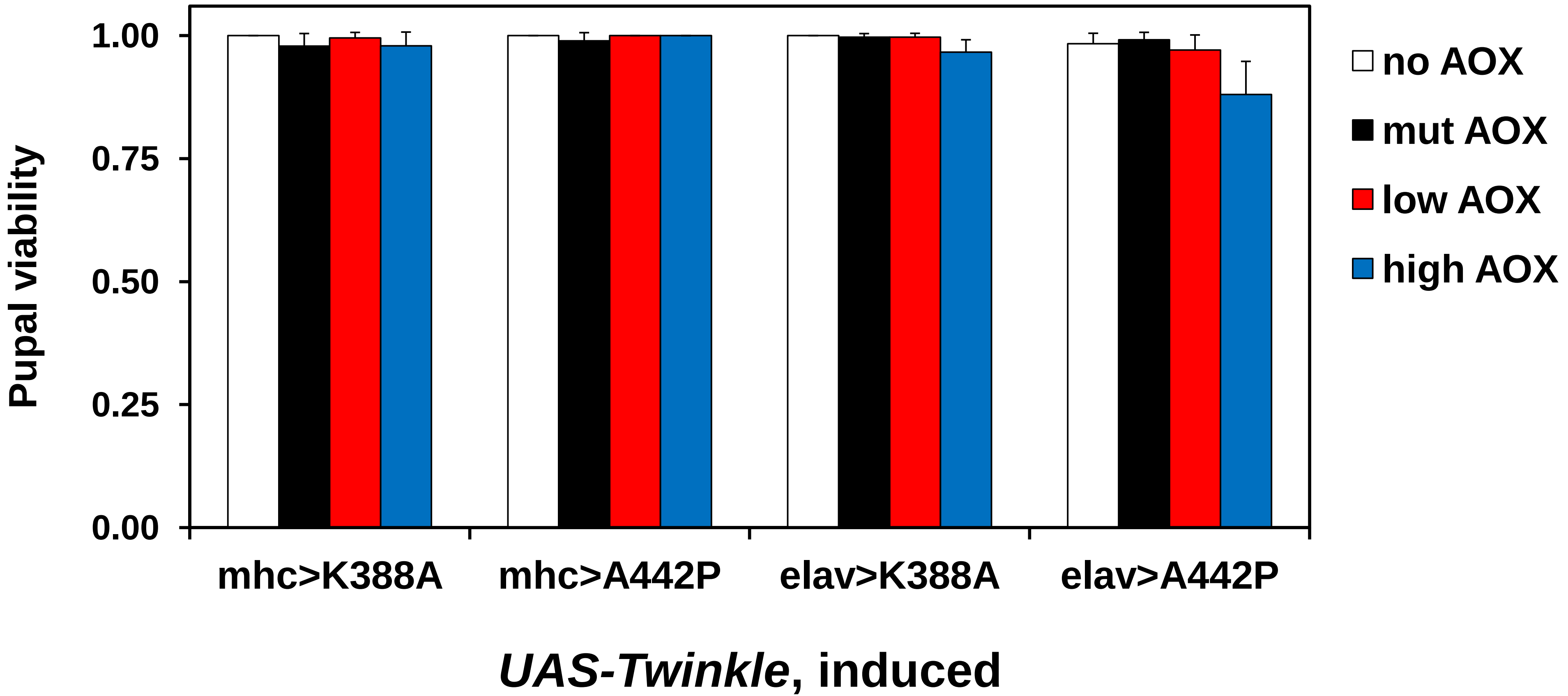

Supplementary Fig. S4 – Rodrigues *et al*.

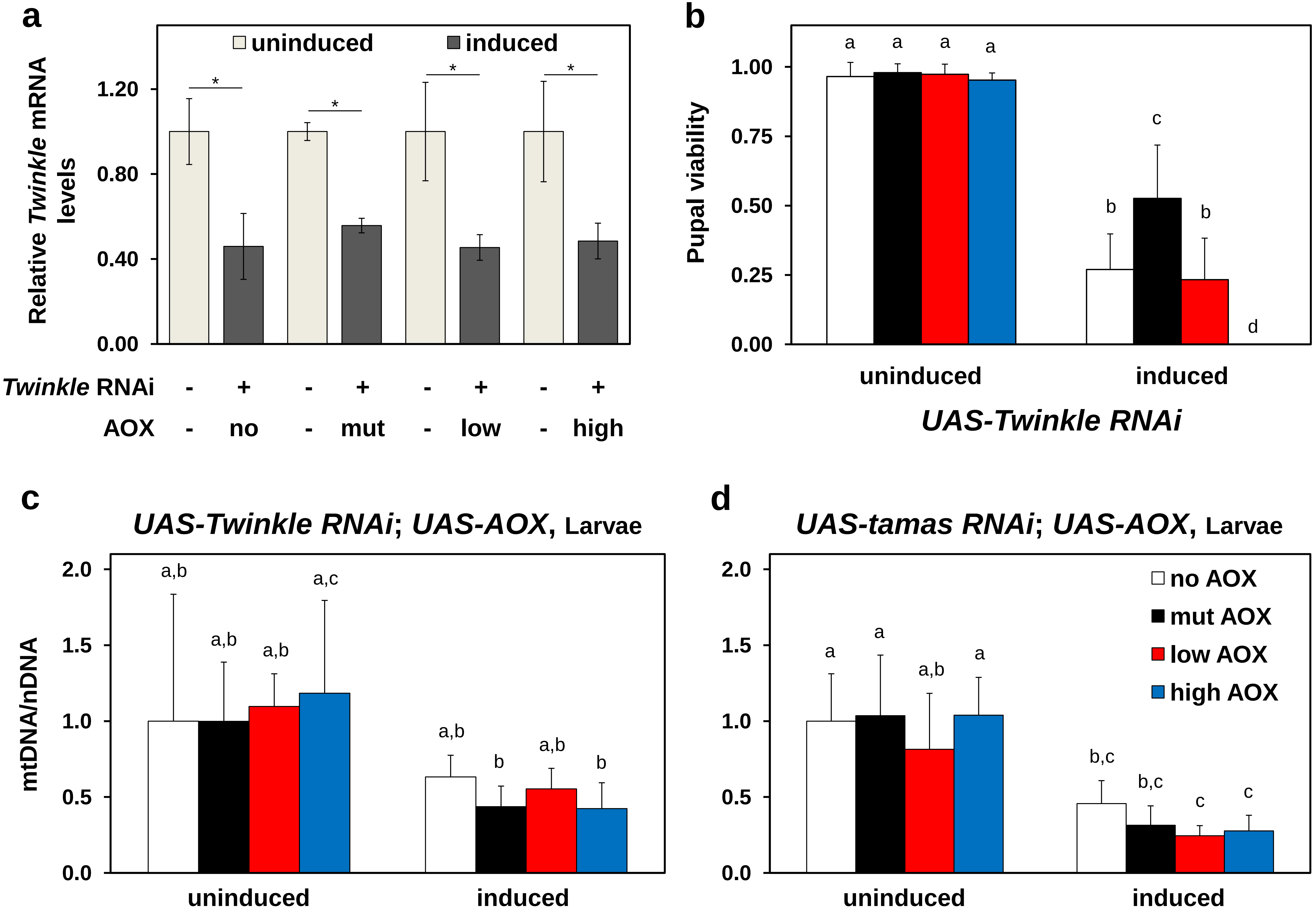

Supplementary Fig. S5 – Rodrigues *et al*.

Crosses involving *tamas Q1009A*:

♀ *tamas Q1009A* / CyO ; *UAS-AOX* / *UAS-AOX* X ♂ *2* / *2* ; *daGAL4* / *daGAL4*

Expected ratio of progeny:		
	0.5	*tamas Q1009A* / *2* ; *UAS-AOX* / *daGAL4*
	0.5	*2* / CyO ; *UAS-AOX* / *daGAL4*

♀ *tamas Q1009A* / CyO ; *UAS-AOX* / *UAS-AOX* X ♂ *2* / *2* ; *3* / *3* (w^{1118})

Expected ratio of progeny:		
	0.5	*tamas Q1009A* / *2* ; *UAS-AOX* / *3*
	0.5	*2* / CyO ; *UAS-AOX* / *3*

♀ *tamas Q1009A* / CyO ; *UAS-AOX* / *UAS-AOX* X ♂ *tamas Q1009A* / CyO ; *daGAL4* / *daGAL4*

Expected ratio of progeny:		
	0.0	*tamas Q1009A* / *tamas Q1009A* ; *UAS-AOX* / *daGAL4*
	1.0	*tamas Q1009A* / CyO ; *UAS-AOX* / *daGAL4*
	0.0	CyO / CyO ; *UAS-AOX* / *daGAL4*

♀ *tamas Q1009A* / CyO ; *UAS-AOX* / *UAS-AOX* X ♂ *tamas Q1009A* / CyO ; *3* / *3*

Expected ratio of progeny:		
	0.0	*tamas Q1009A* / *tamas Q1009A* ; *UAS-AOX* / *3*
	1.0	*tamas Q1009A* / CyO ; *UAS-AOX* / *3*
	0.0	CyO / CyO ; *UAS-AOX* / *3*

- *tamas Q1009A* heterozygotes / AOX expressors (viable controls, induced)
- wild-type *tamas* / AOX expressors (viable controls, *curly wings* phenotype)
- *tamas Q1009A* heterozygotes / AOX non-expressors (viable controls, uninduced)
- wild-type *tamas* / AOX non-expressors (viable controls, *curly wings* phenotype)
- *tamas Q1009A* homozygotes / AOX expressors (experimental progeny, induced)
- homozygotes for CyO balancer (lethal)
- *tamas Q1009A* homozygotes / AOX non-expressors (lethal control, uninduced)

Supplementary Fig. S6 – Rodrigues *et al*.
